# (*meso*-5,5,7,12,12,14-Hexamethyl-1,4,8,11-tetra­aza­cyclo­tetra­deca­ne)nickel(II) bis­[*O*,*O*′-(1,2-phenyl­ene) dithio­phosphate]

**DOI:** 10.1107/S1600536811050951

**Published:** 2011-11-30

**Authors:** Li-Ke Zou, Yan Lu, Jie Cheng, Xi-Yang He, Bin Xie

**Affiliations:** a College of Chemistry and Pharmaceutical Engineering, Sichuan University of Science and Engineering, 643000 Zigong, People’s Republic of China

## Abstract

In the crystal structure of the title compound, [Ni(C_16_H_36_N_4_)](C_6_H_4_O_2_PS_2_)_2_, the Ni^II^ cation is located on a center of inversion and is chelated by the folded tetra­amine macrocycle ligand in a slightly distorted NiN_4_ square-planar geometry. Two symmetry-related *O*,*O*′-(1,2-phenyl­ene)dithio­phosphate anions are located on either side of the Ni^II^ cation, with Ni⋯S of 3.9558 (5) Å, and link to the tetra­amine macrocycle ligand *via* N—H⋯S hydrogen bonding.

## Related literature

For general background to tetra­amine macrocycle compounds, see: Aoki & Kimura (2002 ▶). For the structures of analogous adducts, see: Feng *et al.* (2010 ▶); Lai *et al.* (2011 ▶); Zou *et al.* (2010 ▶). For the synthesis of [Et_3_NH][(*o*-C_6_H_4_O_2_)PS_2_], see: Feng *et al.* (2010 ▶).
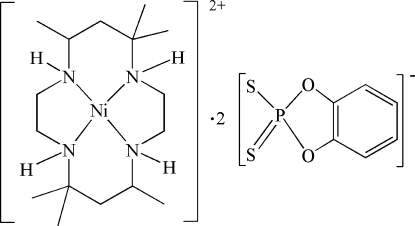


## Experimental

### 

#### Crystal data

[Ni(C_16_H_36_N_4_)](C_6_H_4_O_2_PS_2_)_2_
*M* *_r_* = 749.56
Monoclinic, 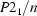

*a* = 9.0012 (15) Å
*b* = 20.500 (3) Å
*c* = 9.6682 (17) Å
β = 101.029 (3)°
*V* = 1751.1 (5) Å^3^
*Z* = 2
Mo *K*α radiation
μ = 0.92 mm^−1^
*T* = 103 K
0.24 × 0.21 × 0.18 mm

#### Data collection

Bruker SMART 1000 CCD area-detector diffractometer
Absorption correction: multi-scan (*SADABS*; Bruker, 2001 ▶) *T*_min_ = 0.809, *T*_max_ = 0.852
9094 measured reflections
3103 independent reflections
2504 reflections with *I* > 2σ(*I*)
*R*_int_ = 0.026

#### Refinement

*R*[*F* ^2^ > 2σ(*F* ^2^)] = 0.036
*wR*(*F* ^2^) = 0.087
*S* = 1.04
3103 reflections
197 parameters
H-atom parameters constrained
Δρ_max_ = 0.40 e Å^−3^
Δρ_min_ = −0.27 e Å^−3^

### 

Data collection: *SMART* (Bruker, 2007 ▶); cell refinement: *SAINT* (Bruker, 2007 ▶); data reduction: *SAINT*; program(s) used to solve structure: *SHELXS97* (Sheldrick, 2008 ▶); program(s) used to refine structure: *SHELXL97* (Sheldrick, 2008 ▶); molecular graphics: *ORTEP-3 for Windows* (Farrugia, 1997 ▶); software used to prepare material for publication: *SHELXL97*.

## Supplementary Material

Crystal structure: contains datablock(s) I, global. DOI: 10.1107/S1600536811050951/xu5399sup1.cif

Structure factors: contains datablock(s) I. DOI: 10.1107/S1600536811050951/xu5399Isup2.hkl

Additional supplementary materials:  crystallographic information; 3D view; checkCIF report

## Figures and Tables

**Table 1:** Selected bond lengths (Å)

Ni1—N1	1.9332 (19)
Ni1—N2	1.9410 (19)

**Table 2:** Hydrogen-bond geometry (Å, °)

*D*—H⋯*A*	*D*—H	H⋯*A*	*D*⋯*A*	*D*—H⋯*A*
N1—H1⋯S2	0.86	2.63	3.444 (2)	158
N2—H2⋯S1^i^	0.86	2.55	3.386 (2)	166

Symmetry code: (i) 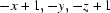.

**Fig. 1.**
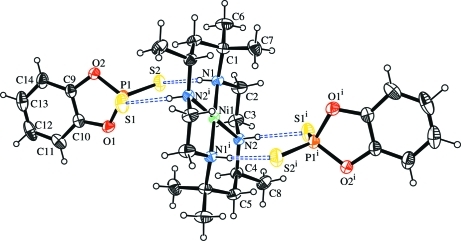
The molecular structure of the title complex, showing the atom-numbering scheme with displacement ellipsoids at 30% probability level. H atoms are represented as small spheres of arbitrary radii and hydrogen-bonds are shown as dashed lines. Atoms with the superscript i are generated by the symmetry operation (-x + 1, -y,-z + 1).

## supplementary crystallographic information

### Comment

Much attentions have been atracted to tetraamine macrocycles as a result of their resemblance to naturally occurring macrocyclic systems (Aoki & Kimura, 2002). In our quest for the potential applications of tetramine macrocycles transition metal complexes as mimetic hydrolases, we have systermly studied their ternary adducts with *O*,*O*'-dialkyldithiophosphate (Feng *et al.*, 2010; Lai *et al.*, 2011; Zou *et al.*, 2010). Herein, we report the structure of an analogue, [Ni(Me_6_[14]aneN_4_)][(*o*-C_6_H_4_O_2_)PS_2_]_2_, where Me_6_[14]aneN_4_ is *meso*-5,5,7,12,12,14-hexamethyl-1,4,8,11-tetraazacyclotetradecane.

The molecular structure of the title adduct is remarkably similar to its analogue, [Cu(Me_6_[14]aneN_4_)][(*o*-C_6_H_4_O_2_)PS_2_]_2_ (Feng *et al.*, 2010). The asymmetric unit consits a centrosymmetric [Ni(Me_6_[14]aneN_4_)]^2+^ dication and two uncoordinated *O*,*O*'-(1,2-phenylene)dithiophosphate anions. The Ni^II^ ion is located on a center of inversion and is chelated by the folded tetraamine macrocycle ligand within slightly distorted NiN_4_ square-plan (Fig.1). Two symmetry related *O*,*O*'-(1,2-phenylene) dithiophosphate anions occupies at psuedo-axial positions with the much longer Ni···S distances of 3.9558 (5) Å. Intermolecular N—H···S hydrogen bondings link the complex cation and pair of anions into three compoment clusters (Table 1). The distorted tetrahedral angles of P atoms range between 93.95 (10) and 121.35 (5)°, illustrating the existence of strain in the *O*,*O*'-(1,2-phenylene)dithiophosphate anions. Two P—S bond lengths are of 1.9383 (12) and 1.9332 (12) Å respectively, which suggests the negative charge is delocalizated over the S1—P1—S2 fragment.

### Experimental

[Et_3_NH][(*o*-C_6_H_4_O_2_)PS_2_] was prepared according to the procedure reported by Feng *et al.* (2010).

A solution of *meso*-5,5,7,12,12,14- hexamethyl-1,4,8,11- tetraazacyclotetradecane dihydrate (0.32 g, 1 mmol) and Ni(Ac)_2_.4H_2_O (0.249 g, 1 mmol) in 25 ml methanol was quickly added to a solution of [Et_3_NH][(*o*-C_6_H_4_O_2_)PS_2_] (0.71 g, 2 mmol) in 25 ml methanol under stirring and refluxed for 6 h. After cooling to room temperature, the precipitate was filtered off, washed successively with methanol and diethyl ether. The obtained orange solid was dissolved in hot methanol and filtered. The filtrate was slowly evaporated at room temperature and orange block crystals suitable for X-ray diffraction studies were obtained after two weeks.

### Refinement

H atoms were placed in calculated positions and treated as riding, with C—H = 0.93–0.98 Å and N—H = 0.86 Å, and refined in a riding mode with *U*_iso_(H) = 1.5*U*_eq_(C,N) for methyl H atoms and imine H atoms and 1.2*U*_eq_(C) for the others.

### Crystal data

**Table:**

[Ni(C_16_H_36_N_4_)](C_6_H_4_O_2_PS_2_)_2_	*F*(000) = 788
*M**_r_* = 749.56	*D*_x_ = 1.422 Mg m^−^^3^
Monoclinic, *P*2_1_/*n*	Mo *K*α radiation, λ = 0.71073 Å
Hall symbol: -P 2yn	Cell parameters from 2892 reflections
*a* = 9.0012 (15) Å	θ = 2.4–24.5°
*b* = 20.500 (3) Å	µ = 0.92 mm^−^^1^
*c* = 9.6682 (17) Å	*T* = 103 K
β = 101.029 (3)°	Block, orange
*V* = 1751.1 (5) Å^3^	0.24 × 0.21 × 0.18 mm
*Z* = 2	

### Data collection

**Table:**

Bruker SMART 1000 CCD area-detector diffractometer	3103 independent reflections
Radiation source: fine-focus sealed tube	2504 reflections with *I* > 2σ(*I*)
graphite	*R*_int_ = 0.026
φ and ω scans	θ_max_ = 25.1°, θ_min_ = 2.0°
Absorption correction: multi-scan (*SADABS*; Bruker, 2001)	*h* = −10→10
*T*_min_ = 0.809, *T*_max_ = 0.852	*k* = −24→21
9094 measured reflections	*l* = −10→11

### Refinement

**Table:**

Refinement on *F*^2^	Primary atom site location: structure-invariant direct methods
Least-squares matrix: full	Secondary atom site location: difference Fourier map
*R*[*F*^2^ > 2σ(*F*^2^)] = 0.036	Hydrogen site location: inferred from neighbouring sites
*wR*(*F*^2^) = 0.087	H-atom parameters constrained
*S* = 1.04	*w* = 1/[σ^2^(*F*_o_^2^) + (0.0391*P*)^2^ + 0.6628*P*] where *P* = (*F*_o_^2^ + 2*F*_c_^2^)/3
3103 reflections	(Δ/σ)_max_ < 0.001
197 parameters	Δρ_max_ = 0.40 e Å^−^^3^
0 restraints	Δρ_min_ = −0.27 e Å^−^^3^

### Special details

**Table:**

Geometry. All e.s.d.'s (except the e.s.d. in the dihedral angle between two l.s. planes) are estimated using the full covariance matrix. The cell e.s.d.'s are taken into account individually in the estimation of e.s.d.'s in distances, angles and torsion angles; correlations between e.s.d.'s in cell parameters are only used when they are defined by crystal symmetry. An approximate (isotropic) treatment of cell e.s.d.'s is used for estimating e.s.d.'s involving l.s. planes.
Refinement. Refinement of *F*^2^ against ALL reflections. The weighted *R*-factor *wR* and goodness of fit *S* are based on *F*^2^, conventional *R*-factors *R* are based on *F*, with *F* set to zero for negative *F*^2^. The threshold expression of *F*^2^ > σ(*F*^2^) is used only for calculating *R*-factors(gt) *etc*. and is not relevant to the choice of reflections for refinement. *R*-factors based on *F*^2^ are statistically about twice as large as those based on *F*, and *R*- factors based on ALL data will be even larger.

### Fractional atomic coordinates and isotropic or equivalent isotropic displacement parameters (Å^2^)

**Table:**

	*x*	*y*	*z*	*U*_iso_*/*U*_eq_	
Ni1	0.5000	0.0000	0.5000	0.03286 (14)	
S1	0.84141 (10)	0.04903 (4)	0.31736 (11)	0.0756 (3)	
S2	0.57109 (9)	0.16238 (5)	0.21370 (10)	0.0726 (3)	
P1	0.78079 (8)	0.13550 (4)	0.24736 (8)	0.0500 (2)	
O1	0.8895 (2)	0.18987 (10)	0.34393 (19)	0.0570 (5)	
O2	0.8521 (2)	0.14989 (10)	0.10467 (19)	0.0585 (5)	
N1	0.3804 (2)	0.03328 (10)	0.3268 (2)	0.0399 (5)	
H1	0.4450	0.0565	0.2935	0.060*	
N2	0.3989 (2)	0.06439 (9)	0.5965 (2)	0.0382 (5)	
H2	0.3256	0.0411	0.6148	0.057*	
C1	0.3140 (3)	−0.01378 (13)	0.2112 (3)	0.0480 (7)	
C2	0.2676 (3)	0.08059 (14)	0.3600 (3)	0.0586 (8)	
H2A	0.2407	0.1116	0.2836	0.070*	
H2B	0.1766	0.0580	0.3729	0.070*	
C3	0.3369 (4)	0.11486 (13)	0.4913 (3)	0.0557 (8)	
H3A	0.2615	0.1409	0.5255	0.067*	
H3B	0.4172	0.1435	0.4741	0.067*	
C4	0.4665 (3)	0.09635 (12)	0.7326 (3)	0.0446 (6)	
H4	0.5371	0.1300	0.7131	0.054*	
C5	0.5544 (3)	0.04792 (14)	0.8347 (3)	0.0519 (7)	
H5A	0.4844	0.0148	0.8546	0.062*	
H5B	0.5927	0.0706	0.9223	0.062*	
C6	0.2265 (4)	0.02438 (17)	0.0854 (3)	0.0718 (10)	
H6A	0.1377	0.0433	0.1104	0.108*	
H6B	0.1972	−0.0046	0.0069	0.108*	
H6C	0.2897	0.0583	0.0600	0.108*	
C7	0.2087 (3)	−0.06079 (15)	0.2663 (4)	0.0655 (9)	
H7A	0.2609	−0.0803	0.3522	0.098*	
H7B	0.1769	−0.0942	0.1975	0.098*	
H7C	0.1217	−0.0375	0.2841	0.098*	
C8	0.3449 (4)	0.12954 (15)	0.7982 (3)	0.0619 (8)	
H8A	0.2989	0.1640	0.7376	0.093*	
H8B	0.3902	0.1473	0.8883	0.093*	
H8C	0.2692	0.0982	0.8102	0.093*	
C9	0.9811 (3)	0.18714 (13)	0.1402 (3)	0.0471 (7)	
C10	1.0029 (3)	0.20927 (12)	0.2761 (3)	0.0468 (7)	
C11	1.1268 (3)	0.24600 (14)	0.3339 (4)	0.0624 (9)	
H11	1.1422	0.2608	0.4265	0.075*	
C12	1.2278 (4)	0.25961 (15)	0.2455 (5)	0.0791 (12)	
H12	1.3141	0.2839	0.2807	0.095*	
C13	1.2051 (4)	0.23869 (18)	0.1089 (5)	0.0787 (11)	
H13	1.2743	0.2497	0.0527	0.094*	
C14	1.0806 (3)	0.20142 (16)	0.0538 (4)	0.0641 (9)	
H14	1.0647	0.1865	−0.0387	0.077*	

### Atomic displacement parameters (Å^2^)

**Table:**

	*U*^11^	*U*^22^	*U*^33^	*U*^12^	*U*^13^	*U*^23^
Ni1	0.0389 (3)	0.0275 (2)	0.0334 (2)	−0.00032 (19)	0.00984 (18)	0.00063 (18)
S1	0.0628 (5)	0.0595 (5)	0.1091 (7)	−0.0099 (4)	0.0279 (5)	0.0137 (5)
S2	0.0503 (5)	0.0852 (7)	0.0857 (6)	−0.0006 (4)	0.0217 (4)	0.0079 (5)
P1	0.0474 (4)	0.0560 (5)	0.0498 (4)	−0.0123 (4)	0.0176 (3)	−0.0053 (3)
O1	0.0628 (12)	0.0608 (13)	0.0503 (11)	−0.0129 (10)	0.0184 (10)	−0.0141 (9)
O2	0.0536 (11)	0.0802 (14)	0.0446 (11)	−0.0208 (11)	0.0167 (9)	−0.0084 (10)
N1	0.0484 (12)	0.0350 (12)	0.0364 (11)	0.0004 (10)	0.0083 (9)	−0.0002 (9)
N2	0.0435 (12)	0.0317 (11)	0.0410 (12)	−0.0022 (9)	0.0126 (9)	−0.0001 (9)
C1	0.0514 (16)	0.0494 (17)	0.0402 (15)	−0.0020 (13)	0.0014 (12)	−0.0059 (12)
C2	0.0638 (19)	0.0555 (18)	0.0521 (17)	0.0238 (15)	−0.0002 (14)	−0.0023 (14)
C3	0.073 (2)	0.0432 (16)	0.0505 (17)	0.0183 (14)	0.0106 (15)	0.0000 (13)
C4	0.0549 (16)	0.0376 (14)	0.0432 (15)	−0.0049 (12)	0.0138 (12)	−0.0072 (12)
C5	0.0651 (18)	0.0538 (17)	0.0369 (15)	−0.0001 (15)	0.0100 (13)	−0.0087 (13)
C6	0.083 (2)	0.079 (2)	0.0445 (18)	0.0131 (19)	−0.0088 (16)	−0.0060 (16)
C7	0.0553 (19)	0.0515 (18)	0.090 (2)	−0.0115 (15)	0.0137 (17)	−0.0112 (17)
C8	0.081 (2)	0.0542 (18)	0.0559 (18)	0.0109 (16)	0.0270 (16)	−0.0090 (14)
C9	0.0394 (15)	0.0453 (16)	0.0571 (17)	0.0003 (12)	0.0103 (13)	0.0099 (13)
C10	0.0436 (15)	0.0351 (14)	0.0619 (18)	0.0019 (12)	0.0109 (13)	0.0032 (13)
C11	0.0501 (17)	0.0376 (16)	0.091 (2)	0.0022 (14)	−0.0069 (17)	−0.0060 (15)
C12	0.0405 (18)	0.0412 (19)	0.152 (4)	−0.0033 (15)	0.010 (2)	0.015 (2)
C13	0.050 (2)	0.069 (2)	0.123 (3)	0.0075 (18)	0.031 (2)	0.040 (2)
C14	0.0521 (19)	0.075 (2)	0.070 (2)	0.0051 (17)	0.0218 (16)	0.0247 (17)

### Geometric parameters (Å, °)

**Table:**

Ni1—N1	1.9332 (19)	C4—C8	1.526 (4)
Ni1—N1^i^	1.9332 (19)	C4—H4	0.9800
Ni1—N2^i^	1.9410 (19)	C5—C1^i^	1.514 (4)
Ni1—N2	1.9410 (19)	C5—H5A	0.9700
S1—P1	1.9383 (12)	C5—H5B	0.9700
S2—P1	1.9332 (12)	C6—H6A	0.9600
P1—O1	1.6497 (19)	C6—H6B	0.9600
P1—O2	1.6554 (19)	C6—H6C	0.9600
O1—C10	1.374 (3)	C7—H7A	0.9600
O2—C9	1.377 (3)	C7—H7B	0.9600
N1—C2	1.483 (3)	C7—H7C	0.9600
N1—C1	1.511 (3)	C8—H8A	0.9600
N1—H1	0.8600	C8—H8B	0.9600
N2—C3	1.483 (3)	C8—H8C	0.9600
N2—C4	1.491 (3)	C9—C10	1.368 (4)
N2—H2	0.8600	C9—C14	1.368 (4)
C1—C5^i^	1.514 (4)	C10—C11	1.373 (4)
C1—C7	1.518 (4)	C11—C12	1.390 (5)
C1—C6	1.532 (4)	C11—H11	0.9300
C2—C3	1.480 (4)	C12—C13	1.366 (5)
C2—H2A	0.9700	C12—H12	0.9300
C2—H2B	0.9700	C13—C14	1.377 (5)
C3—H3A	0.9700	C13—H13	0.9300
C3—H3B	0.9700	C14—H14	0.9300
C4—C5	1.512 (4)		
			
N1—Ni1—N1^i^	180.00 (13)	C5—C4—C8	110.4 (2)
N1—Ni1—N2^i^	93.33 (8)	N2—C4—H4	108.0
N1^i^—Ni1—N2^i^	86.67 (8)	C5—C4—H4	108.0
N1—Ni1—N2	86.67 (8)	C8—C4—H4	108.0
N1^i^—Ni1—N2	93.33 (8)	C4—C5—C1^i^	117.0 (2)
N2^i^—Ni1—N2	180.00 (9)	C4—C5—H5A	108.1
O1—P1—O2	93.95 (10)	C1^i^—C5—H5A	108.1
O1—P1—S2	110.90 (9)	C4—C5—H5B	108.1
O2—P1—S2	109.30 (9)	C1^i^—C5—H5B	108.1
O1—P1—S1	108.88 (9)	H5A—C5—H5B	107.3
O2—P1—S1	109.02 (9)	C1—C6—H6A	109.5
S2—P1—S1	121.33 (5)	C1—C6—H6B	109.5
C10—O1—P1	109.87 (17)	H6A—C6—H6B	109.5
C9—O2—P1	109.47 (16)	C1—C6—H6C	109.5
C2—N1—C1	112.8 (2)	H6A—C6—H6C	109.5
C2—N1—Ni1	109.51 (16)	H6B—C6—H6C	109.5
C1—N1—Ni1	119.43 (16)	C1—C7—H7A	109.5
C2—N1—H1	105.0	C1—C7—H7B	109.5
C1—N1—H1	106.1	H7A—C7—H7B	109.5
Ni1—N1—H1	102.4	C1—C7—H7C	109.5
C3—N2—C4	109.58 (19)	H7A—C7—H7C	109.5
C3—N2—Ni1	107.08 (15)	H7B—C7—H7C	109.5
C4—N2—Ni1	124.98 (15)	C4—C8—H8A	109.5
C3—N2—H2	109.1	C4—C8—H8B	109.5
C4—N2—H2	105.2	H8A—C8—H8B	109.5
Ni1—N2—H2	99.7	C4—C8—H8C	109.5
N1—C1—C5^i^	106.9 (2)	H8A—C8—H8C	109.5
N1—C1—C7	109.4 (2)	H8B—C8—H8C	109.5
C5^i^—C1—C7	112.7 (2)	C10—C9—C14	121.7 (3)
N1—C1—C6	109.4 (2)	C10—C9—O2	112.5 (2)
C5^i^—C1—C6	108.4 (2)	C14—C9—O2	125.8 (3)
C7—C1—C6	110.0 (2)	C9—C10—C11	121.9 (3)
C3—C2—N1	107.6 (2)	C9—C10—O1	112.3 (2)
C3—C2—H2A	110.2	C11—C10—O1	125.8 (3)
N1—C2—H2A	110.2	C10—C11—C12	115.8 (3)
C3—C2—H2B	110.2	C10—C11—H11	122.1
N1—C2—H2B	110.2	C12—C11—H11	122.1
H2A—C2—H2B	108.5	C13—C12—C11	122.5 (3)
C2—C3—N2	107.4 (2)	C13—C12—H12	118.8
C2—C3—H3A	110.2	C11—C12—H12	118.8
N2—C3—H3A	110.2	C12—C13—C14	120.6 (3)
C2—C3—H3B	110.2	C12—C13—H13	119.7
N2—C3—H3B	110.2	C14—C13—H13	119.7
H3A—C3—H3B	108.5	C9—C14—C13	117.5 (3)
N2—C4—C5	111.2 (2)	C9—C14—H14	121.3
N2—C4—C8	111.0 (2)	C13—C14—H14	121.3
			
O2—P1—O1—C10	−12.84 (19)	C4—N2—C3—C2	−178.4 (2)
S2—P1—O1—C10	−125.24 (16)	Ni1—N2—C3—C2	43.3 (3)
S1—P1—O1—C10	98.80 (17)	C3—N2—C4—C5	−170.2 (2)
O1—P1—O2—C9	12.24 (19)	Ni1—N2—C4—C5	−41.2 (3)
S2—P1—O2—C9	126.01 (16)	C3—N2—C4—C8	66.5 (3)
S1—P1—O2—C9	−99.28 (17)	Ni1—N2—C4—C8	−164.52 (18)
N2^i^—Ni1—N1—C2	172.52 (18)	N2—C4—C5—C1^i^	60.0 (3)
N2—Ni1—N1—C2	−7.48 (18)	C8—C4—C5—C1^i^	−176.3 (2)
N2^i^—Ni1—N1—C1	40.32 (19)	P1—O2—C9—C10	−8.4 (3)
N2—Ni1—N1—C1	−139.68 (19)	P1—O2—C9—C14	171.1 (2)
N1—Ni1—N2—C3	−19.90 (17)	C14—C9—C10—C11	−1.1 (4)
N1^i^—Ni1—N2—C3	160.10 (17)	O2—C9—C10—C11	178.4 (2)
N1—Ni1—N2—C4	−149.9 (2)	C14—C9—C10—O1	179.5 (2)
N1^i^—Ni1—N2—C4	30.1 (2)	O2—C9—C10—O1	−1.0 (3)
C2—N1—C1—C5^i^	167.1 (2)	P1—O1—C10—C9	10.0 (3)
Ni1—N1—C1—C5^i^	−62.1 (3)	P1—O1—C10—C11	−169.3 (2)
C2—N1—C1—C7	−70.6 (3)	C9—C10—C11—C12	0.4 (4)
Ni1—N1—C1—C7	60.1 (3)	O1—C10—C11—C12	179.7 (3)
C2—N1—C1—C6	49.9 (3)	C10—C11—C12—C13	0.8 (5)
Ni1—N1—C1—C6	−179.32 (19)	C11—C12—C13—C14	−1.5 (5)
C1—N1—C2—C3	169.0 (2)	C10—C9—C14—C13	0.5 (4)
Ni1—N1—C2—C3	33.5 (3)	O2—C9—C14—C13	−179.0 (3)
N1—C2—C3—N2	−50.3 (3)	C12—C13—C14—C9	0.8 (5)

Symmetry codes: (i) −*x*+1, −*y*, −*z*+1.

### Hydrogen-bond geometry (Å, °)

**Table:**

*D*—H···*A*	*D*—H	H···*A*	*D*···*A*	*D*—H···*A*
N1—H1···S2	0.86	2.63	3.444 (2)	158
N2—H2···S1^i^	0.86	2.55	3.386 (2)	166

Symmetry codes: (i) −*x*+1, −*y*, −*z*+1.
